# *HOXB13* is a susceptibility gene for prostate cancer: results from the International Consortium for Prostate Cancer Genetics (ICPCG)

**DOI:** 10.1007/s00439-012-1229-4

**Published:** 2012-10-12

**Authors:** Jianfeng Xu, Ethan M. Lange, Lingyi Lu, Siqun L. Zheng, Zhong Wang, Stephen N. Thibodeau, Lisa A. Cannon-Albright, Craig C. Teerlink, Nicola J. Camp, Anna M. Johnson, Kimberly A. Zuhlke, Janet L. Stanford, Elaine A. Ostrander, Kathleen E. Wiley, Sarah D. Isaacs, Patrick C. Walsh, Christiane Maier, Manuel Luedeke, Walther Vogel, Johanna Schleutker, Tiina Wahlfors, Teuvo Tammela, Daniel Schaid, Shannon K. McDonnell, Melissa S. DeRycke, Geraldine Cancel-Tassin, Olivier Cussenot, Fredrik Wiklund, Henrik Grönberg, Ros Eeles, Doug Easton, Zsofia Kote-Jarai, Alice S. Whittemore, Chih-Lin Hsieh, Graham G. Giles, John L. Hopper, Gianluca Severi, William J. Catalona, Diptasri Mandal, Elisa Ledet, William D. Foulkes, Nancy Hamel, Lovise Mahle, Pal Moller, Isaac Powell, Joan E. Bailey-Wilson, John D. Carpten, Daniela Seminara, Kathleen A. Cooney, William B. Isaacs, International Consortium for Prostate Cancer Genetics

**Affiliations:** 1 Data Coordinating Center for the ICPCG, Wake Forest University School of Medicine, Winston-Salem, NC USA; 2 Center for Cancer Genomics, Wake Forest University School of Medicine, Winston-Salem, NC USA; 3 University of Michigan ICPCG Group, University of Michigan Medical School, Ann Arbor, MI USA; 4 Departments of Genetics and Biostatistics, University of North Carolina, Chapel Hill, NC USA; 5 Mayo Clinic ICPGC Group, Mayo Clinic, Rochester, MN USA; 6 Department of Laboratory Medicine and Pathology, Mayo Clinic, Rochester, MN USA; 7 University of Utah ICPCG Group, University of Utah School of Medicine, Salt Lake City, UT USA; 8 Department of Medicine, University of Utah School of Medicine, Salt Lake City, UT USA; 9 Department of Internal Medicine, University of Michigan Medical School, Ann Arbor, MI USA; 10 Fred Hutchinson Cancer Research Center (FHCRC) ICPCG Group, Seattle, WA USA; 11 Division of Public Health Sciences, FHCRC, Seattle, WA USA; 12 Cancer Genetics Branch, National Human Genome Research Institute, NIH, Bethesda, MD USA; 13 Johns Hopkins University ICPCG Group, Baltimore, MD USA; 14 Department of Urology, Johns Hopkins Medical Institutions, Johns Hopkins Hospital, Marburg 115, 600 North Wolfe Street, Baltimore, MD 21287 USA; 15 University of Ulm ICPCG Group, University of Ulm, Ulm, Germany; 16 Department of Urology, University of Ulm, Ulm, Germany; 17 Institute of Human Genetics, University of Ulm, Ulm, Germany; 18 University of Tampere ICPCG Group, University of Tampere and Fimlab Laboratories, Tampere, Finland; 19 Institute of Biomedical Technology/BioMediTech, University of Tampere and Fimlab Laboratories, Tampere, Finland; 20 Department of Medical Biochemistry and Genetics, University of Turku, Turku, Finland; 21 Department of Urology, Tampere University Hospital, Tampere, Finland; 22 Department of Health Sciences Research, Mayo Clinic, Rochester, MN USA; 23 CeRePP ICPCG Group, Paris, France; 50 Department of Urology, APHP, Hospital Tenon, Paris, France; 24 CeRePP UPMC University, Paris, France; 25 Karolinska ICPCG Group, Karolinska Institutet, Stockholm, Sweden; 26 Department of Medical Epidemiology and Biostatistics, Karolinska Institutet, Stockholm, Sweden; 27 ACTANE (Anglo/Canadian/Texan/Australian/Norwegian/EU Biomed) Consortium ICPCG Group, Surrey, UK; 28 Institute of Cancer Research and Royal Marsden NHS Foundation Trust, Surrey, UK; 29 Strangeways Laboratory, Department of Oncology, Centre for Cancer Genetic Epidemiology, University of Cambridge, Cambridge, UK; 30 BC/CA/HI ICPCG Group, Stanford School of Medicine, Stanford, CA USA; 31 Department of Health Research and Policy, Stanford School of Medicine, Stanford, CA USA; 32 Stanford Comprehensive Cancer Center, Stanford School of Medicine, Stanford, CA USA; 33 Department of Urology and Department of Biochemistry and Molecular Biology, University of Southern California, Los Angeles, CA USA; 34 Cancer Epidemiology Centre, Cancer Council Victoria, Melbourne, Australia; 35 Centre for Molecular, Environmental, Genetic and Analytical Epidemiology, University of Melbourne, Melbourne, Australia; 36 Northwestern University ICPCG Group, Chicago, IL USA; 37 Northwestern University Feinberg School of Medicine, Chicago, IL USA; 38 Louisiana State University ICPCG Group, New Orleans, LA USA; 39 Department of Genetics, Louisiana State University Health Sciences Center, New Orleans, LA USA; 40 Program in Cancer Genetics, Departments of Oncology and Human Genetics, McGill University, Montreal, QC Canada; 51 Research Institute of the McGill University Health Centre, Montreal, QC Canada; 41 The Norwegian Radium Hospital, Oslo, Norway; 42 African American Hereditary Prostate Cancer ICPCG Group, Detroit, MI USA; 43 Karmanos Cancer Institute, Wayne State University, Detroit, MI USA; 44 Inherited Disease Research Branch, National Human Genome Research Institute, NIH, Bethesda, MD USA; 45 Genetic Basis of Human Disease Research Division, Translational Genomics Research Institute, Phoenix, AZ USA; 46 National Cancer Institute, NIH, Bethesda, MD USA

## Abstract

Prostate cancer has a strong familial component but uncovering the molecular basis for inherited susceptibility for this disease has been challenging. Recently, a rare, recurrent mutation (G84E) in *HOXB13* was reported to be associated with prostate cancer risk. Confirmation and characterization of this finding is necessary to potentially translate this information to the clinic. To examine this finding in a large international sample of prostate cancer families, we genotyped this mutation and 14 other SNPs in or flanking *HOXB13* in 2,443 prostate cancer families recruited by the International Consortium for Prostate Cancer Genetics (ICPCG). At least one mutation carrier was found in 112 prostate cancer families (4.6 %), all of European descent. Within carrier families, the G84E mutation was more common in men with a diagnosis of prostate cancer (194 of 382, 51 %) than those without (42 of 137, 30 %), *P* = 9.9 × 10^−8^ [odds ratio 4.42 (95 % confidence interval 2.56–7.64)]. A family-based association test found G84E to be significantly over-transmitted from parents to affected offspring (*P* = 6.5 × 10^−6^). Analysis of markers flanking the G84E mutation indicates that it resides in the same haplotype in 95 % of carriers, consistent with a founder effect. Clinical characteristics of cancers in mutation carriers included features of high-risk disease. These findings demonstrate that the *HOXB13* G84E mutation is present in ~5 % of prostate cancer families, predominantly of European descent, and confirm its association with prostate cancer risk. While future studies are needed to more fully define the clinical utility of this observation, this allele and others like it could form the basis for early, targeted screening of men at elevated risk for this common, clinically heterogeneous cancer.

**Electronic supplementary material:**

The online version of this article (doi:10.1007/s00439-012-1229-4) contains supplementary material, which is available to authorized users.

## Introduction

By sequencing coding regions of more than 200 genes in a previously identified region of linkage at 17q21–22 (Lange et al. 2003; Gillanders et al. 2004; Xu et al. 2005; Lange et al. 2007; Cropp et al. 2011) a rare but recurrent mutation (G84E) in *HOXB13* was recently identified in four of 94 probands from prostate cancer families. (Ewing et al. 2012) The mutation co-segregated with prostate cancer in these four families and was found to be significantly more common among 5,083 unrelated prostate cancer patients (1.4 %) than control subjects (0.1 %) of European descent (*p* = 8.5 × 10^−7^) leading to odds ratio (OR) estimates of tenfold or more. In this initial report, the frequency of the mutation was higher in prostate cancer patients with early-onset disease (age at diagnosis ≤55 years old, 2.2 %) or with a positive family history (2.2 %), and most common in patients with both of these features (3.1 %). If confirmed, these findings provide support for the concept that rare, moderately penetrant mutations as well as common, low-penetrance prostate cancer risk-associated variants identified from genome-wide association studies (GWAS) (Gudmundsson et al. 2007a, b, 2008, 2009; Yeager et al. 2007, 2009; Thomas et al. 2008; Eeles et al. 2008, 2009; Sun et al. 2008; Xu et al. 2010; Kote-Jarai et al. 2011a; Takata et al. 2010; Akamatsu et al. 2012; Haiman et al. 2011) both contribute to prostate cancer risk. The identification and characterization of genetic variants reproducibly associated with substantial increases in prostate cancer risk would provide enhanced ability to identify men most likely to benefit from early disease screening.

Prostate cancer demonstrates wide differences in incidence and mortality across populations within the United States and throughout the world. In an attempt to confirm and expand the observations of Ewing et al. (2012), we examined the frequency of *HOXB13* G84E mutations in prostate cancer families across different ancestries and geographic regions. We genotyped this mutation and other known variants in *HOXB13* in 2,443 hereditary prostate cancer families recruited by members of the International Consortium for Prostate Cancer Genetics (ICPCG), a large NCI-funded collaborative resource for studies of genetic susceptibility for hereditary prostate cancer.

## Subjects and methods

### Study population

The ICPCG study cohort has been described in detail previously (Schaid and Chang 2005; Xu et al. 2005). Fifteen groups participated in the present study, including those from Europe [Finland (Tampere University), Sweden (Karolinska Institute), UK (Institute of Cancer Research and Royal Marsden NHS Foundation Trust, University of Cambridge, ACTANE), Germany (University of Ulm), and France (CeRePP)], North America (Fred Hutchinson Cancer Research Center, Johns Hopkins Hospital, Louisiana State University, Mayo Clinic, McGill University, Northwestern University, Stanford University, University of Michigan, and University of Utah), and Australia (University of Melbourne) (Supplementary Table 1).

Each ICPCG group recruited its study population via different methods of pedigree ascertainment and utilized different methods to confirm prostate cancer diagnosis. In this study, men were considered “affected” if their prostate cancer diagnosis was confirmed by either medical records or death certificates. All other men were assigned as “unknown phenotype.” A total of 2,443 families were included in the study, including 6,422 affected men and 1,902 men without a prostate cancer diagnosis (unknown), and 1,803 women whose DNA samples were available (Supplementary Table 1). Research protocols and study documentation were approved by each group’s Institutional Review Board.

### SNPs selection and genotyping

Five mutations in the *HOXB13* gene, selected from the original paper of Ewing et al. (2012) and the ESP database (Exome Variant Server, NHLBI Exome Sequencing Project, Seattle, WA, USA (URL: http://evs.gs.washington.edu/EVS/) [1/2012]) were genotyped in the ICPCG dataset, including G84E (c.251G > A, rs138213197), T105I (c.314C > T, rs140492479), R217C (c.649C > T, rs13945791), R229G (c.685C > G), and T253P (c.757A > C). In addition, ten polymorphic SNPs (rs890435, rs2326017, rs7212669, rs8064938, rs3809773, rs1054072, rs8556, rs3809771, rs4793980, rs3110601) flanking the *HOXB13* gene and spanning 108,191 base pairs (bp) from 46,719,399 to 46,827,590 (Build 37) were genotyped to estimate allele frequencies and haplotypes. The G84E mutation, due to a change in the second position of codon 84 (GGA → GAA), results in a nonconservative substitution in a conserved putative protein–protein binding motif of HOXB13 (Ewing et al. 2012).

Genotyping was performed using the MassARRAY iPLEX (Sequenom, Inc., San Diego, CA, USA). Duplicates and negative controls were included in each 96-well plate to ensure quality control (QC). Genotyping was performed by technicians blinded to the sample status. The average concordance rate was 99.7 % for 6,300 genotypes among QC duplicates.

### Statistical methods

Frequency of the G84E mutation was determined at either family level or individual level. At a family level, the proportion of families with at least one G84E mutation carrier was determined for the entire set as well as for each ICPCG group. The difference in the proportion among different ICPCG groups was tested using Chi-square with a degree of freedom (*df*) of 14. At an individual level, the proportion of G84E mutation carriers was compared among men with a diagnosis of prostate cancer (affecteds) and the remaining men within the families (unknowns). The difference of G84E mutation carrier rate between affected and unknown men was tested based on a marginal model that accounts for relatedness of subjects within families using generalized estimating equations (GEE). An exchangeable working correlation matrix was assumed.

A family-based association test was performed to test association of the G84E mutation and other SNPs with prostate cancer by assessing over-transmission of alleles from parents to affected offspring using the computer program FBAT (Xu et al. 2002). Empirical variance test statistics were used to account for the correlation of transmitted alleles among multiple affected individuals in the same family.

Haplotypes of each individual based on these 15 SNPs were estimated using Genehunter-plus (Kruglyak et al. 1996) and PLINK (Purcell et al. 2007). The haplotypes with the highest likelihood were selected. For subjects whose inferred haplotypes were different based on these two methods, manual inspection was performed to resolve the difference, with priority given to haplotypes based on linkage disequilibrium among markers in this study population.

## Results

Among five previously observed mutations in *HOXB13* (Ewing et al. 2012) two were observed in this study—R217C (rs13945791) and G84E (rs138213197). The rare R217C variant was found one time each in two families of European descent and did not co-segregate with prostate cancer. The G84E mutation was found in 283 subjects from 112 families of European descent, including 194 men with prostate cancer (Table 1). This represented 4.6 % of all 2,443 prostate cancer families and 4.8 % of 2,298 prostate cancer families of European descent. The proportion of families with at least one G84E mutation carrier differed significantly across the 15 ICPCG groups (*P* = 9.4 × 10^−8^). The proportion was highest in families from the Nordic countries of Finland (22.4 %) and Sweden (8.2 %) and lower in North America (0–6.1 %) and Australia (2.6 %). The G84E mutation was not found in families of any other race or ethnicity, including those of African (*N* = 58), Ashkenazi Jewish (*N* = 46), or other descent (*N* = 28). Obviously, larger numbers of families of these and other races and ethnicities will need to be examined to more fully characterize the population distribution of this mutation.

**Table 1:** G84E mutation of *HOXB13* in prostate cancer families of International Consortium for Prostate Cancer Genetics (ICPCG)

	No. of families		No. of families with G84E carriers (%)		Subjects in families with at least one G84E carrier					
					Affected		Unknown (Men)		Unknown (Women)	
	All	European descent	All	European descent	*N*	No. of G84E carriers (%)	*N*	No. of G84E carriers (%)	*N*	*N* of G84E carriers (%)
Europe										
Finland, University of Tampere	76	76	17 (22.4 %)	17 (22.4 %)	54	37 (69 %)	69	22 (31 %)	97	29 (30 %)
Sweden, Umea University	110	110	9 (8.2 %)	9 (8.2 %)	17	13 (76 %)	15	5 (33 %)	13	4 (31 %)
Germany, University of Ulm	378	378	13 (3.4 %)	13 (3.4 %)	21	19 (90 %)	1	0 (0 %)	2	0 (0 %)
UK, ACTANE	145	142	5 (3.4 %)	5 (3.5 %)	12	7 (58 %)	1	0 (0 %)	1	0 (0 %)
France, CeRePP	159	156	2 (1.3 %)	2 (1.3 %)	5	3 (60 %)	1	0 (0 %)	0	0
North America										
BC/CA/HI	98	83	6 (6.1 %)	6 (7.2 %)	20	12 (60 %)	7	1 (14 %)	7	1 (14 %)
Fred Hutchinson Cancer Research Center	255	241	14 (5.5 %)	14 (5.8 %)	45	25 (56 %)	14	5 (36 %)	16	2 (13 %)
Johns Hopkins Hospital^a^	234	176	5 (2.1 %)	5 (2.8 %)	20	14 (70 %)	7	2 (29 %)	10	4 (40 %)
MAYO Clinic	185	185	6 (3.2 %)	6 (3.2 %)	15	10 (67 %)	2	0 (0 %)	0	0
University of Michigan^a^	317	282	11 (3.5 %)	11 (3.9 %)	36	26 (72 %)	13	4 (31 %)	5	2 (40 %)
McGill University	18	17	1 (5.9 %)	1 (5.9 %)	2	2 (100 %)	0	0	0	0
North Western University	33	32	0 (0 %)	0 (0 %)	0	0	0	0	0	0
University of Utah	348	348	21 (6 %)	21 (6 %)	132	23 (17 %)	6	2 (33 %)	11	3 (27 %)
Louisiana State University	10	10	0 (0 %)	0 (0 %)	0	0	0	0	0	0
Australia										
Australia	77	73	2 (2.6 %)	2 (2.7 %)	3	3 (100 %)	1	1 (100 %)	3	2 (67 %)
Total	2,443	2,309	112 (4.6 %)	112 (4.9 %)	382	194 (51 %)	137	42 (31 %)	165	47 (28 %)
Total^a^	1,892	1,851	96 (5.0 %)	96 (5.2 %)	326	154 (47 %)	117	36 (31 %)	150	41 (27 %)

^a^A subset of families from these centers were included in the original discovery report (Ewing et al. 2012). These total values reflect the results obtained after omitting all families from these two centers

In the 112 families with at least one G84E mutation carrier, the mutation was found in both affected and unaffected men. However, the carrier rate was significantly higher in affected men (194 of 382, 51 %) than other men in these families (i.e. men of unknown status [(42 of 137, 31 %), *p* = 9.9 × 10^−8^]) (Table 1). Using a statistical test that considered the relatedness of subjects within carrier families, the odds ratio (OR) for prostate cancer was 4.42 [95 % confidence interval (CI) 2.56–7.64] for the G84E mutation carriers. We repeated our analyses excluding families from the University of Michigan and Johns Hopkins Hospital, some of which were included in the initial report describing *HOXB13* as a prostate cancer susceptibility gene (Ewing et al. 2012). In particular, the former study included *HOXB13* G84E genotype data from only the youngest prostate cancer case in a subset of University of Michigan and Johns Hopkins Hospital families. The carrier rate in ICPCG families remained significantly higher in affected men (154 of 326, 47 %) than unknown men [(36 of 117, 31 %), *P* = 3.3 × 10^−6^] and the OR for prostate cancer was 4.3 [95 % confidence interval (CI) 2.32–7.96] for the G84E mutation carriers after excluding all families from these two institutions (Table 1).

A mixed pattern of co-segregation of the G84E mutation with prostate cancer was found in these 112 families. While complete co-segregation was found in 34 families, incomplete co-segregation was more commonly observed, revealing genetic heterogeneity (affected but not carriers) and incomplete penetrance of the mutation (carriers but unaffected men).

We also examined transmission of G84E mutation and alleles of other genotyped SNPs at the region in all 2,443 families using a family-based association test (Table 2). The risk allele (A) corresponding to the G84E mutation was observed to be transmitted significantly more often than expected from parents to affected sons (*P* = 6.5 × 10^−6^). A significant result was also observed when all families from the University of Michigan and Johns Hopkins Hospital were removed from this analysis (*P* = 1.2 × 10^−4^) (Supplementary Table 2), strongly indicating the G84E mutation is associated with prostate cancer risk.

**Table 2:** Family-based association test for SNPs at *HOXB13* region in ICPCG families

Chr	Position	rs#	Gene	Mutation	Rare allele	Allele frequency	No. of informative families	S-E(S)^a^	Var(S)	*Z*	*P*
17	46,719,399	rs890435	Intergenic		G	0.41	509	−7.38	243.77	−0.47	0.64
17	46,720,565	rs2326017	Intergenic		T	0.33	496	3.10	248.24	0.20	0.84
17	46,727,289	rs7212669	Intergenic		G	0.10	244	−4.89	107.42	−0.47	0.64
17	46,780,829	rs8064938	Intergenic		A	0.16	353	−6.12	136.42	−0.52	0.60
17	46,784,039	rs3809773	Intergenic		A	0.33	485	1.42	245.54	0.10	0.93
17	46,799,812	rs1054072	PRAC		C	0.47	518	−13.41	268.62	−0.82	0.41
17	46.804,250		HOXB13	T253P		0	0	N/A	N/A	N/A	N/A
17	46,804,322		HOXB13	R229G	G	0.0001	1	−0.40	0.16	−1.00	0.32
17	46,804,358	rs139475791	HOXB13	R217C	A	0.0001	2	−1.60	1.36	−1.37	0.17
17	46,805,590	rs8556	HOXB13		T	0.15	342	−10.77	145.60	−0.89	0.37
17	46,805,642	rs140492479	HOXB13	T105I	A	0.0001	2	1.64	1.41	1.38	0.17
17	46,805,705	rs138213197	HOXB13	G84E	A	0.02	38	17.50	15.07	4.51	6.53E−06
17	46,807,919	rs3809771	5′		G	0.06	171	−8.92	64.24	−1.11	0.27
17	46,813,531	rs4793980	5′		T	0.16	306	2.22	116.03	0.21	0.84
17	46,827,590	rs3110601	5′		C	0.12	274	−7.46	114.18	−0.70	0.49

Based on an FBAT analysis of 2,437 pedigrees (10,217 nuclear families; 40,246 subjects)

^a^S-E(S) is the statistical score for the observed number of rare allele transmissions minus the statistical score for the expected number of transmissions

To assess association in our family set while adjusting for variable pedigree structures, we randomly selected one affected man (proband) in the second generation from each of 2,443 pedigrees and then counted the number of G84E carriers among probands, first-relatives, and second-degree relatives or higher (Table  3). The G84E mutation carrier rate among probands was 2.8 %. Among the first-degree relatives, the carrier rate was significantly higher in affected men (75 %) than in those with an unknown phenotype (48 %), *P* = 0.002, OR = 4.26 (95 % CI 1.69–10.75). Among the second-degree relatives or higher, the carrier rate was also significantly higher in affected men (58 %) than in unknown men (23 %), *P* = 0.004, OR = 4.81 (95 % CI 1.64–14.12).

**Table 3:** G84E *HOXB13* mutation carriers among randomly selected affected probands and their relatives

Proband G84E Carrier	G84E carriers in first-degree relatives				G84E carriers in second-degree relatives or higher			
	Affected	Unknown	OR (95 % CI)	*P* value	Affected	Unknown	OR (95 % CI)	*P* value
Yes (51)	56/75 (74.7 %)	16/34 (47.6 %)	4.26 (1.69–10.75)	0.002	11/19 (57.9 %)	9/39 (23.1 %)	4.81 (1.64–14.12)	0.004
No (1,755)	21/2,502 (0.8 %)	3/759 (0.4 %)	2.31 (0.82–6.51)	0.11	15/973 (1.5 %)	6/651 (0.9 %)	2.21 (0.39–12.71)	0.37

The prostate cancer patients who carried the mutation had a wide spectrum of clinical disease, including cancers with high risk of disease progression (Table 4), as indicated by moderate to poor tumor differentiation (tumor grade of Gleason score 7 or higher) in over one-third of the cases with available data, and over one-quarter having non-organ confined disease at diagnosis (tumor stage T3 or higher). The mean age at diagnosis of carriers was 62.8 years. In comparison, the mean age at diagnosis for the 6,172 prostate cancer patients without the mutation was 64.4 years (*P* = 0.04; relatedness of subjects within families was considered). The mean age at last contact of G84E carriers without a prostate cancer diagnosis was 56.3.

**Table 4:** Clinicopathologic variables of prostate cancers in *HOXB13* G84E carriers

	No. of patients	% of patients
Tumor grade (Gleason Score)		
≤6	67	63.2
7	32	30.2
8	4	3.8
≥9	3	2.8
Tumor stage		
T1c or lower	47	39.2
T2	41	34.2
T3 or higher	32	26.7
Metastasis at diagnosis		
Yes	4	3.1
Serum PSA level at diagnosis		
≤10	49	48.0
11–20	25	24.5
≥20	28	27.5
Age at diagnosis		
≤55	24	18.6
56–80	105	81.4
≥80	0	0.0
Death from prostate cancer		
Yes	9	7.0

Finally, to assess a potential founder effect for the G84E mutation, we estimated haplotypes based on the 15 genotyped SNPs in this region. The mutation (allele A) of G84E was predicted to be on eight different haplotypes. However, 95 % (269 out of 283) of the occurrences were predicted to be on a single rare haplotype (frequency of 2 %). Among the 269 G84E mutation carriers predicted to carry the common haplotype, 83 were from Finland while the remaining were from 12 other ICPCG groups. One individual from Finland was homozygous for all 15 markers, allowing unambiguous assignment of the haplotype. This individual was diagnosed with moderately differentiated (Gleason 7), clinically localized prostate cancer at age 60.

We note that the genotype data for all 269 G84E mutation carriers were consistent with a single shared haplotype spanning the 15 genotyped SNPs (i.e. there were no SNPs that had homozygous genotypes for opposite alleles among the 269 carriers) and it is possible that with additional genotype data the most likely haplotype configuration for G84E carriers would be a single founder haplotype.

## Discussion

By evaluating germline mutations of the *HOXB13* gene in 2,433 prostate cancer families from the ICPCG, this study confirmed the observation that the G84E mutation is significantly associated with prostate cancer in subjects of European descent with family history of the disease. The results remained significant when families used in the original report were not included in the analysis, providing independent confirmation of the original finding. Although there is a large degree of variability in the number of individuals sampled per pedigree in the ICPCG, approximately 5 % of prostate cancer families had at least one member with the G84E mutation. These results are consistent with the hypothesis that *HOXB13* G84E is a prostate cancer susceptibility allele that significantly increases the risk of prostate cancer.

The search for hereditary prostate cancer genes has been challenging due to a number of factors including the late-onset nature of the disease and the high background rate of sporadic disease in the general population. Although rare variants of other genes such as *RNASEL* (Carpten et al. 2002), *MSR1* (Xu et al. 2002), and *ELAC2* (Tavtigian et al. 2001) have been previously identified in prostate cancer families and proposed as prostate cancer susceptibility alleles, follow-up studies have not supported their candidacy. On the other hand, mutations in *BRCA2* have been reproducibly associated with prostate cancer risk (Edwards et al. 2003), but their frequency is low in prostate cancer families (Agalliu et al. 2007; Kote-Jarai et al. 2011b).

More recently, GWAS studies have led to the identification of over 40 prostate cancer risk-associated SNPs that have been replicated in multiple study populations. These variants are common in the general population (5 % or higher), confer low risk with ORs, typically in the range of 1.1–1.4 (Gudmundsson et al. 2007a, b, 2008, 2009; Yeager et al. 2007, 2009; Thomas et al. 2008; Eeles et al. 2008, 2009; Sun et al. 2008; Xu et al. 2010; Kote-Jarai et al. 2011a; Takata et al. 2010; Akamatsu et al. 2012; Haiman et al. 2011), and have been estimated to account for ~25 % of the risk associated with a positive family history (Kote-Jarai et al. 2011a). Although more common prostate cancer risk-associated variants are likely to be identified in the future, rare variants with larger effects have been proposed as an alternative mechanism to account for ‘missing inheritance’ (Iyengar and Elston 2007; Bodmer and Bonilla 2008). In this respect, the establishment of a rare and moderate- to high-penetrance mutation in *HOXB13* as a prostate cancer susceptibility allele provides empirical evidence for this alternative hypothesis. Indeed, like colorectal and breast cancer, at least some significant fraction of prostate cancer risk is conferred by this class of coding sequence variants.

The estimated frequency of the *HOXB13* G84E mutation in prostate cancer families is influenced by the number of individuals in any given family as well as family structure. For example, some extended families, particularly in the Utah collection, have more than 100 subjects and have multiple affected generations. Similarly, estimated ORs for G84E in relation to prostate cancer risk are impacted by the mixed degrees of relatedness among relatives, as the covariance matrices used in the GEE models do not explicitly account for family structure. The analysis presented in Table 3 was designed to provide better odds ratio estimates for first- and second-degree relatives of G84E carriers. Of interest, the carrier rate was lower among second-degree affected relatives (58 %) compared with first-degree affected relatives (75 %), suggesting the presence of genetic heterogeneity across families. The OR estimates from our analyses should be interpreted only in the context of the current study. We note that the odds ratios are calculated based on many “controls” that have limited phenotype information; most have not been screened for disease or screening results are missing. Further, familial controls not currently affected by prostate cancer are more likely to develop disease in the future compared with randomly selected men from the general population given the strong history of disease in these families. Finally, our familial cases are more likely to carry moderate to high penetrance risk alleles compared with typical unselected prostate cancer cases. Large population-based studies that include screened men will be necessary to obtain more accurate measures of G84E mutation frequency and penetrance. As we observed, the frequency of G84E mutations are likely population specific.

Our results implicate a geographical frequency gradient of the G84E mutation across the European continent, with the mutation being more common in Nordic countries, notably Finland. This finding highlights the strength of the current study as family-based association methods provide the strongest protection against type I error due to population stratification. It remains to be seen how various analytic methods (e.g. those based on principal components that capture the major sources of genetic variation between subjects across common genetic variants) will protect against population stratification when analyzing uncommon genetic variants that disproportionately occur in specific European-derived populations in case–control settings.

In summary, analysis of the large ICPCG family collection establishes the *HOXB13* G84E allele as a reproducible risk factor for prostate cancer. Our identification of a common haplotype among the majority of *HOXB13* G84E carriers indicates that there is a founder effect with a higher frequency of the mutant allele in Nordic populations. Additional studies using population-based case–control and/or familial samples will be useful to define the penetrance of this mutation, which will have important clinical implications for families that carry the G84E mutation.

## Electronic supplementary material

Below is the link to the electronic supplementary material.

Supplementary material 1 (DOCX 346 kb)

Supplementary material 2 (DOCX 614 kb)
