# Supplementary figures and images for: HOXB13 is a susceptibility gene for prostate cancer: results from the International Consortium for Prostate Cancer Genetics (ICPCG)
Source: Hum Genet. 2012 Oct 12;132(1):5–14. doi: 10.1007/s00439-012-1229-4 (PMC3535370; doi:10.1007/s00439-012-1229-4)

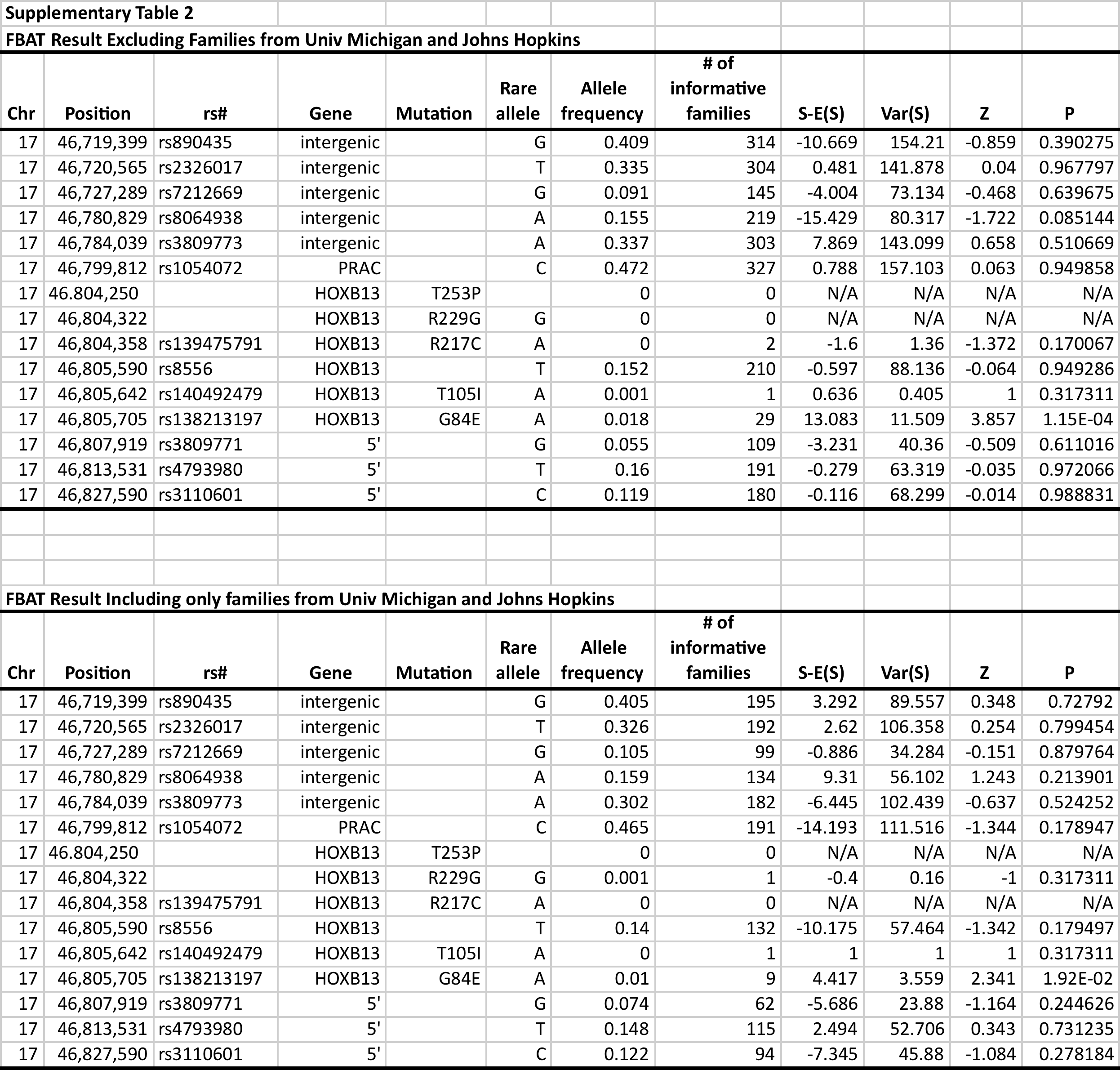

Supplement: Supplementary file 2 — Supplementary material 2 (DOCX 614 kb) [file 439_2012_1229_MOESM2_ESM.docx]
